# Exposure to Environmental Toxicants and Pathogenesis of Amyotrophic Lateral Sclerosis: State of the Art and Research Perspectives

**DOI:** 10.3390/ijms140815286

**Published:** 2013-07-24

**Authors:** Francesca Trojsi, Maria Rosaria Monsurrò, Gioacchino Tedeschi

**Affiliations:** 1 Department of Medical, Surgical, Neurological, Metabolic and Aging Sciences, Second University of Naples, Piazza Miraglia 2, Naples 80138, Italy; E-Mails: mrmonsurro@hotmail.com (M.R.M.); gioacchino.tedeschi@unina2.it (G.T.); 2 Neurological Institute for Diagnosis and Care “Hermitage Capodimonte”, Via Cupa delle Tozzole 2, Naples 80131, Italy

**Keywords:** amyotrophic lateral sclerosis, environmental toxicants, cyanobacteria, heavy metals, lead, methylmercury, selenium, pesticides, organophosphates, neurodegeneration

## Abstract

There is a broad scientific consensus that amyotrophic lateral sclerosis (ALS), a fatal neuromuscular disease, is caused by gene-environment interactions. In fact, given that only about 10% of all ALS diagnosis has a genetic basis, gene-environmental interaction may give account for the remaining percentage of cases. However, relatively little attention has been paid to environmental and lifestyle factors that may trigger the cascade of motor neuron degeneration leading to ALS, although exposure to chemicals—including lead and pesticides—agricultural environments, smoking, intense physical activity, trauma and electromagnetic fields have been associated with an increased risk of ALS. This review provides an overview of our current knowledge of potential toxic etiologies of ALS with emphasis on the role of cyanobacteria, heavy metals and pesticides as potential risk factors for developing ALS. We will summarize the most recent evidence from epidemiological studies and experimental findings from animal and cellular models, revealing that potential causal links between environmental toxicants and ALS pathogenesis have not been fully ascertained, thus justifying the need for further research.

## 1. Introduction

Amyotrophic lateral sclerosis (ALS) is an idiopathic, fatal neurodegenerative disease of the human motor system characterized by degeneration of both cortical and brainstem or spinal motor neurons [1]. Clinical presentation of ALS classically includes muscle weakness localized to a single limb and/or bulbar district which becomes progressively widespread, leading to quadriparesis, dysarthria and dysphagia. The final stage consists in respiratory failure and death.

During recent years the concept of ALS is changing from a clinically defined syndrome characterized by upper and lower motor neurons degeneration to a multisystem disorder which shows significant overlaps with frontotemporal dementia [2]. Neuropathological and genetic findings have led to hypothesize a new model for ALS pathogenesis strongly influenced by accumulation of protein aggregates in the cytoplasm of degenerating neuronal cells [3]. In fact, emerging evidence supports the notion that misfolding of ALS-linked proteins may be significantly involved in triggering the neurodegenerative process [4]. In particular, mutant types of superoxide dismutase 1 (SOD1) and TAR DNA binding protein 43 kDa (TDP-43) are key molecules involved in the pathogenesis of familial (fALS) and sporadic ALS (sALS), respectively [5,6].

It is becoming increasingly clear that ALS is a polygenic disease with a variable penetrance [2]. Moreover, given that sALS represents approximately 90% of ALS cases, it has been hypothesized that exposure to environmental pollutants may interact with individual genetic susceptibility playing a remarkable role in the pathogenesis of the disease. A growing list of potential environmental risk factors for ALS has been proposed, including exposure to cyanobacteria, heavy metals, pesticides, intense physical activity, head injury, cigarette smoking, electromagnetic fields (EMF) and electrical shocks [7–19], although at present there is no ascertained causal link between environmental toxicants and ALS pathogenesis.

The role of toxic agents has received renewed attention because of the emerging hypothesis that exposure to some environmental toxicants might have played a relevant role in triggering the neurodegenerative process in genetically predisposed subjects belonging to some communities with an increased risk of ALS, such as the Chamorro indigenous people of Guam and the Veterans of the 1991 Gulf War [20–22]. The high incidence rate of ALS among Chamorro Guamanians has proved to be the result of the cumulative consumption of cycad flour and flying fox [20,23]. In particular, β-*N*-methylamino-l-alanine (BMAA), a known neurotoxin produced by most cyanobacterial taxa and identified in cycad seeds [20], has been detected in a bound form in hair and wing membrane of flying fox, regular part of Chamorro diet [24]. Another ALS endemic focus similar to Guam has been later recognized in the Kii Peninsula of Japan. In both Pacific foci the clinical presentation has changed over the last 50 years into a broader spectrum of neurodegenerative diseases, known as ALS/Parkinsonism-dementia complex (ALS/PDC) [25,26].

With regard to another community with an increased risk of ALS, among 1991 Gulf War veterans the age of onset of ALS was found to be significantly earlier [27,28], inducing to speculate that they may have a “Persian Gulf” variant of ALS with atypical clinical features, such as shorter ventilator-free survival than not-deployed military personnel [28]. Several potential etiologic agents were hypothesized for Gulf War syndrome, including genetic predisposition alone (e.g., paroxonase genes variants) [29,30] or in combination with environmental exposures or stress-related responses [21,22,31–36]. Specifically, with regard to potential sources of neurotoxicity, Miranda *et al.* [21] suggested an association between particular locations of service in the Persian Gulf, such as Khamisiyah (Iraq), where soldiers might be mostly exposed to nerve agents (e.g., sarin and cyclosarin) from the destruction of the munitions and facilities, and the subsequent occurrence of ALS. However, while results about a potential association between exposure to environmental toxicants in Khamisiyah and risk of developing neurological disorders have been proved conflicting [31–34], exposure to cyanobacteria from dust from desert cryptogamic crust or from drinking or swimming in cyanobacteria-contaminated water has been found significantly related to Gulf War syndrome [35,36].

In this review we aim to give an overview of recent advances about potential toxic etiologies of ALS, especially those emerged in the last five years, underlining current knowledge about cyanobacteria, heavy metals and pesticides. Furthermore, we will cover most of the intriguing evidence on gene-environment interactions as probable trigger of the neurodegenerative process, in line with a multifactorial model for ALS pathogenesis according to which genetic and environmental components should be complementary to form a mosaic picture.

## 2. BMAA and Cyanobacteria

The non-protein amino acid BMAA, produced by cyanobacteria living symbiotically in the coralloid roots of cycad trees (*Cycas micronesica*) and proved to be neurotoxic to both rodents and primates [37–40], has recently received renewed attention as environmental risk factor for sALS [19]. In truth, the failure to identify a genetic marker for Guam ALS/PDC [41,42] and the finding of decreased incidence of disease with increased travel from Guam to other countries [43] have suggested that an environmental toxin might be responsible of Guam neurodegenerative disorders. Particularly, at the end of World War II incidence, prevalence and mortality rates of ALS in the Chamorro people of Guam were found to be 50 to 100 times those of ALS elsewhere [44]. The use of cycad flour to make tortillas, soups and dumplings by the native Chamorro people [45,46] led to the suggestion that cycad consumption could be the cause of a progressive and irreversible neurological syndrome frequently reported in death certificates from Guam [46], and thus the cycad hypothesis was born. Afterwards, this hypothesis was abandoned because it was revealed that more than 80% of BMAA may be removed from cycad seeds with a single wash, thereby making it impossible to consume toxic quantities [47]. However, further evidence from studies on life-style of Guam Chamorros showed that human exposure to BMAA may occur through the consumption of flying foxes or fruit bats (*Pteropus mariannus*) and other animals, such as pigs and deer, feeding on cycad seeds [20,48]. It was discovered that BMAA bioaccumulated in *Pteropus mariannus* [49,50] and was detected in brain tissues of Chamorro people affected by ALS/PDC [48,49]. Specifically, to support the bioaccumulation hypothesis, Banack and Cox [48] examined also dried skin sample of flying foxes from museum specimens (given that native species of flying foxes were almost extinct in Guam), revealing BMAA concentrations equivalent (per weight) to up to 1014 kg of processed cycad flour.

**Figure 1:**
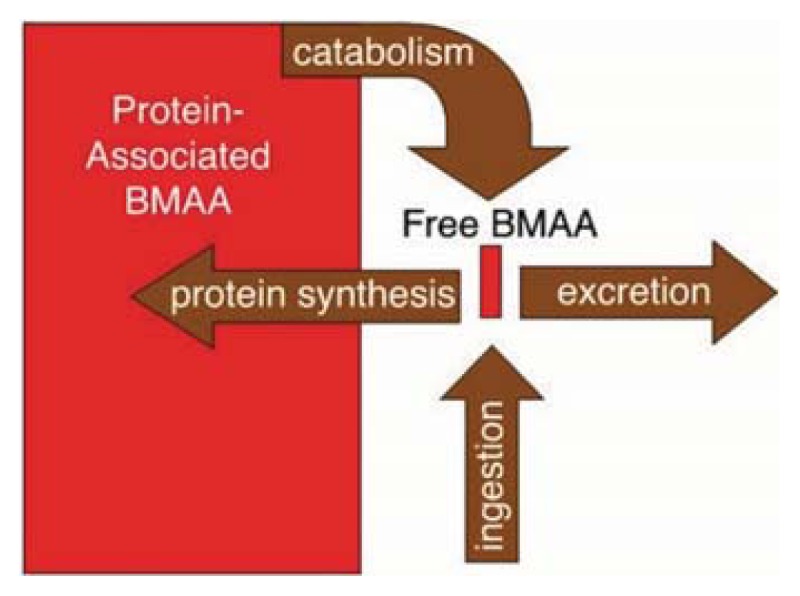
Schematic representation of the endogenous neurotoxic reservoir of Beta-*N*-methylamino-l-alanine (BMAA) that slowly and continuously releases the neurotoxin into cerebral tissue through protein metabolism (Source: Murch *et al.* [24]).

From the pathogenetic point of view, Murch *et al.* [24] found that chronic dietary intake of BMAA has led to its misincorporation into brain tissues of six out of six Chamorro people, all of whom died of ALS/PDC; additionally, two Canadians died of Alzheimer’s disease. Particularly, BMAA was detected in a bound form, which was supposed to function as an endogenous neurotoxic reservoir, accumulating and subsequently releasing BMAA during digestion and protein metabolism (Figure 1). Therefore, Murch *et al.* [24] hypothesized that the endogenous neurotoxic reservoir might slowly release free BMAA within brain tissues, thereby inducing a neurotoxic injury sustained over years or even decades. In support of this hypothesis came the more recent findings from animal and cellular models, which demonstrated that BMAA was misincorporated into brain proteins, producing protein misfolding, aggregates and cell death [51,52]. Increased brain levels of BMAA were later confirmed by Pablo *et al.* [53], who also found similar BMAA levels in brains of Florida patients with ALS, but not in brains of healthy subjects.

Insights from studies on animal models have confirmed the probable involvement of BMAA into neurodegenerative process. Intriguingly, Karllson *et al.* [39] revealed that exposure of neonatal rats to BMAA may produce early hippocampal cell death and learning and memory impairments in adulthood. More recently, it was demonstrated in the same animal model that developmental exposure to high doses of BMAA produced changes in the expression of histones, calcium- and calmodulin-binding proteins, and guanine nucleotide-binding proteins, provoking severe lesions in the adult hippocampus with neuronal degeneration, cell loss, calcium deposits, and astrogliosis [40].

At present, there is no way to measure the BMAA content in living human brains to relate these measures to the risk of developing ALS or other neurodegenerative diseases. Therefore, epidemiological research could be useful to rely upon exposure to cyanobacteria as a surrogate for BMAA exposure. To this purpose, Bradley *et al.* [19] underlined the usefulness of ad-hoc questionnaires and direct collection of environmental samples to assess indirectly the exposure to cyanotoxins. Further, with regard to the possible source of exposure to BMAA, it is to take into account that over 90% of cyanobacterial species may produce BMAA [54], as well as the free-living marine *Nostoc* species [55], proving that BMAA could be detected ubiquitously. In fact, bioaccumulation of BMAA has been identified in aquatic species such as zooplankton, fish, mussels and oysters in the Baltic Sea [56], as well as in food chains in South Florida [57], in waters of lakes in China, Michigan, New Hampshire and Finland [58–61], and in desert dust from the Middle East [35,36].

Potential role of genetic susceptibility to neurotoxicity of BMAA has been explored by several studies, without identifying, however, any candidate genes [62]. Speculatively, genetic differences in absorption, distribution and metabolism of cyanotoxins might influence individual susceptibility to develop a neurodegenerative process. An example of such genetic susceptibility concerns the polymorphisms in the cholesterol transport apolipoprotein E (APOE) gene, given that in a mouse model cycad toxicity seems to be in part regulated by apoE variants [63]. However, Sundar *et al.* [64] found that in a population of 600 Chamorros from Guam the ɛ4 and ɛ2 APOE allele frequencies were not significantly different between ALS/PDC cases and controls. Conversely, microtubule-associated protein tau gene was proven to be associated with risk for Guam neurodegenerative disorders [64]. Future intriguing genetic targets may be mutations or polymorphisms of the genes encoding the neutral l-1 amino acid transporter for BMAA [65], which regulates transport of BMAA across the blood-brain barrier, and the seryl-tRNA synthetase [66], which modulates incorporation of BMAA into neuronal proteins.

## 3. Heavy Metals

It has been shown that metals may induce pathological conditions when deficient or accumulated to toxic levels. Currently, the potential role of several heavy metals into the molecular mechanisms that lead to degeneration of motoneurons has been widely explored and only partially characterized [11,15,16,18]. Specifically, most insights concern lead, mercury, selenium, copper and zinc, although most heavy metals, also including aluminum and cadmium, have been investigated as potential risk factors for ALS [67–69].

### 3.1. Lead

Several studies of ALS patients and human mutant SOD1 G93A transgenic mice showed that lead (Pb) exposure was associated with greater survival [70–72]. In fact, greater tibia and blood Pb levels were found to be associated with increased survival of ALS patients, inducing to postulate that Pb exposure may delay disease progression [73]. In agreement with these results, a preliminary case-control study on a population of 34 ALS patients from the South of Italy showed that blood Pb levels were not significantly increased in patients compared with controls and did not correlate with disease severity [74]. These findings are rather questionable given the large amount of data in the literature indicating that Pb is a neurotoxic and not a neuroprotective agent [75–79] and has showed significant higher levels in cerebrospinal fluid and blood from ALS patients compared with controls [67,69,80]. More recently longer survival was detected in case of chronic exposure of SOD1 G93A transgenic mice to low Pb levels. It was hypothesized that Pb exposure could have stimulated an increased expression of vascular endothelial growth factor in the spinal anterior horns of the transgenic mice, thereby inducing a decreased activation of astrocytes with neuroprotective effects [81].

Conversely, Kamel *et al.* [82] revealed that some polymorphisms in the genes for δ-aminolevulinic acid dehydratase (ALAD), which resulted significantly associated with bone Pb levels in ALS patients compared with controls, may affect ALS risk through a mechanism related to internal Pb exposure.

One potential explanation for the above mentioned conflicting results is that Pb exposure is only part of the “equation” which should be investigated. In fact, epigenetic changes (e.g., DNA modifications that do not change directly the DNA sequence but lead to alterations in the transcription and translation processes), which may be induced by Pb exposure, have recently received growing attention in order to explain the possible role played by Pb [18,83].

### 3.2. Mercury

With regard to human exposure, methylmercury (MeHg) is the most important organic mercury (Hg) compound. Diet, through fish and derivates, is its dominant source, but occupational exposure is also considerable [84].

Epidemiological and case-control studies have shown an association between Hg exposure and risk of developing ALS [85–87]. In particular, Schwarz *et al.* [88] observed clinical symptoms similar to those typical of ALS in a patient with long-term accidental Hg exposure. Moreover, Sienko *et al.* [59] identified a population of ALS patients in a small Wisconsin community, which was characterized by large consumption of fresh-caught fish, a species that showed high levels of Hg when it was caught in Lake Michigan.

About the role of occupational exposure, Barber [89] discovered neurological changes that resembled those found in ALS in some employees of a mercuric oxide manufacturing plant. However, those results were in disagreement with Pamphlett and Waley [90], who found no significant differences in inorganic Hg levels in upper and lower motor neurons of sALS patients compared with controls.

Some retrospective case-control studies found no association between Hg and other heavy metals in the pathogenesis of ALS [91,92]. However, evidence provided by animal and cultural studies seems to suggest that Hg exposure might be implicated in the etiology of ALS. In fact, it has been found that a single dose of mercuric chloride (HgCl_2_) in mice may cause deposition of Hg in upper and lower motor neurons [93,94]. Johnson and Atchison [95] recently revealed that mice over expressing mutant human SOD1 G93A, long-term exposed to low levels of MeHg, showed an anticipation of the onset of ALS phenotype. This supports the hypothesis that exposure to a toxic metal such as MeHg may hasten the onset of ALS in case of underlying genetic predisposition for ALS.

More recently, interesting insights derived from *in vitro* studies using cellular cultures. Intriguingly, it was found that low doses of MeHg can interfere with the secretion of some pro-inflammatory cytokines like interleukin-6 (IL-6) from microglial cells, modifying the interaction between microglia and astrocytes [96]. This impaired mechanism seems to provide a plausible link between MeHg exposure and impaired glial function, which has been proved to be involved in several neurodegenerative conditions [97–99].

With regard to Hg interfering with epigenetic mechanisms, it has been observed that cysteine-*S*-MeHg conjugates are structurally similar to methionine, and are suspected to function as molecular mimics of methionine at certain amino acid transporters [100]. Given that methionine plays a role in providing methyl groups to DNA methyltransferase, it is worth considering that molecular mimicry of methionine might allow organic Hg conjugates to interfere with DNA methylation [101].

### 3.3. Selenium

Excessive exposure to the metalloid selenium (Se), a trace element with both toxicological and nutritional properties, has been implicated in the etiology of ALS. Two remarkable epidemiological investigations, which documented an increased risk of ALS in populations resident in seleniferous areas, have allowed for supporting a probable causal link between Se exposure and ALS risk. The former study was carried out by Kilness and Hochberg [102], who reported four ALS cases in a scarcely populated county of west-central South Dakota, characterized by high incidence of selenosis in farm animals. Therefore, the association between the high Se environmental levels and the numerous ALS cases detected in this area was supposed to be causal. The latter epidemiological analysis was performed by Vinceti *et al.* [103] in the Northern Italy municipality of Reggio Emilia, where high levels of Se were detected in the waters of the two wells, which were the source of municipal tap water from 1972 to 1988. A follow-up study, carried out for eleven years in a cohort of 5182 inhabitants who drank for at least five years the high-Se tap water, revealed an increased ALS risk in this municipality (*i.e.*, four newly diagnosed ALS cases, compared the 0.64 expected ones).

Further support to an association between Se and ALS came from Yang *et al.* [104] who identified clusters of patients with neurological signs and symptoms affecting motor function in seleniferous areas of China, although this study did not include a detailed report on the relation between risk of developing degeneration of the motor system and degree of Se exposure.

More recent experimental and laboratory models have found that the central nervous system (CNS) and muscles may be damaged by Se overexposure, thereby yielding a biological plausibility to an association between Se and ALS risk [105,106]. Specifically, Se, in both organic and inorganic compounds, has been proved to play a contributory role in several pathogenetic mechanisms involved in the neurotoxic process (*i.e*., inhibition of prostaglandin D synthase, succinic dehydrogenase, acetylcholine esterase and sodium/potassium ATPase in the brain, increase in dopamine and its metabolites, and induction of seizures) [106–110].

The most interesting “biological evidence” supporting a Se-ALS relation came from veterinary medicine observations. In particular, Se may alter neuromuscular functions in rabbits, rats and chicks [111–114], whilst it showed a specific toxicity to motor neurons in swine and cattle [115,116]. Accidental Se intoxication has been found to selectively damage motor neurons in swine, provoking a bilateral focal poliomalacia of spinal cord ventral horns. Several motor symptoms, characterized by walking disturbances or paralysis and death from respiratory failure, have also been revealed in cattle from seleniferous areas [117]. These motor abnormalities have been experimentally reproduced in Se-exposed steers, showing that they were associated with polioencephalomalacia [118].

**Figure 2:**
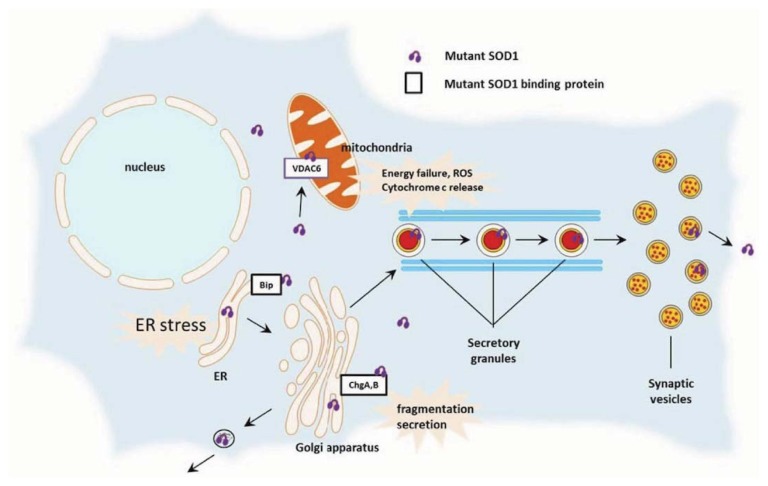
Aberrant subcellular localization of mutant SOD1 protein and its effect on amyotrophic lateral sclerosis (ALS) pathogenesis: mutant SOD1 interacts with several binding proteins (*i.e.*, endoplasmic reticulum chaperone protein GRP78/Bip, voltage-dependent anion channel 6 or VDCA6, and chromogranin A or ChgA and B or ChgB), resulting in cell death when abnormally redistributed (Source: Ido *et al.* [4]).

Recently, another mechanism possibly linking Se to ALS etiology was recognized in the nematode *Caenorhabditis elegans* by Estevez *et al.* [119] who found that Se-induced oxidative stress led to decreased cholinergic signaling and degeneration of cholinergic motor neurons through depletion of glutathione. Furthermore, Maraldi *et al.* [120] examined the role of exposure to low Se levels in triggering oxidative damage and apoptosis in a human neuronal cell line. This *in vitro* study showed that (i) a decreased viability was found in neuroblastoma cells, treated with sodium selenite, sodium selenate and seleno-methionine (0.1, 0.5 and 0.5 mM, respectively) for 24 h, unlike kidney or prostatic cells, and apoptosis was inducted in neuroblastoma cells by poly-ADP-ribose-polymerase degradation and caspase activation; (ii) during Se treatment, an increased ratio between reactive oxygen species (ROS) and reactive nitrogen species (RNS) occurred; (iii) Se exposure induced SOD1 translocation into mitochondria similarly to what described in ALS [4,121] (Figure 2). In particular, the oxidant properties of Se species might lead to a compensatory response of the cell, probably ineffective to antagonize the onset of apoptosis. Thus, Se exposure may considerably influence SOD1 accumulation into mitochondria, a somatic feature occurring in neurons during ALS pathogenesis (Figure 2). These findings might indicate a toxic effect of Se even at low concentrations, suggesting that current upper limits of Se intake through diet and overall exposure may be too high and should be restated.

### 3.4. Zinc and Copper

Zinc (Zn) has been shown to be substantially implicated in selective neuronal death after ischemia [122]. Moreover, the accumulation of Zn triggering neurodegeneration could be avoided by Zn chelators [123].

Recently, it has been demonstrated that Zn exposure may significantly contribute to oxidative neurotoxicity in spinal cord motor neurons, and may be related to chronic neurodegeneration. In fact, under the activation of calcium permeable α-amino-3-hydroxy-5-methy-4-isoxazole propionate (AMPA)/kainate channels in cultured neuronal cells, Zn exposure for 1 hour induced a significant increase of 4-hydroxynonenal expression and a significant decrease of glutamate transporter GLT-1 expression, producing a strong increase in ROS generation and a significant decrease of spinal cord neuron survival [124]. Moreover, in cultured cortical neurons from SOD1 G93A transgenic mice the exposure to sublethal concentrations of Zn has been found to enhance *N*-methyl-d-aspartate receptor (NMDAR)-mediated excitotoxicity [125].

Recently, high daily intake of Zn (75 and 375 mg/kg/day) has been reported to increase the death of mutant SOD1 G93A transgenic mice [126]. Nonetheless, moderate supplementation of Zn (e.g., about 12 mg/kg/day) has been found to delay death in SOD1 G93A mice by 11 days compared to mice on a Zn-deficient diet [127]. In fact, Ermilova *et al.* [127] revealed that large amounts of Zn may compete with copper (Cu) absorption, leading to Cu deficiency and inhibition of Cu-dependent ceruloplasmin, which can induce a lethal anemia. By contrast, the supplementation of Zn dosage with Cu has been found to prevent the early death from Zn treatment alone. Thus, Ermilova *et al.* [127] supported a role for moderate levels of dietary Zn potentially protecting against the toxicity of ALS-associated SOD1 mutation and this protective effect does not result from depleting Cu.

**Figure 3:**
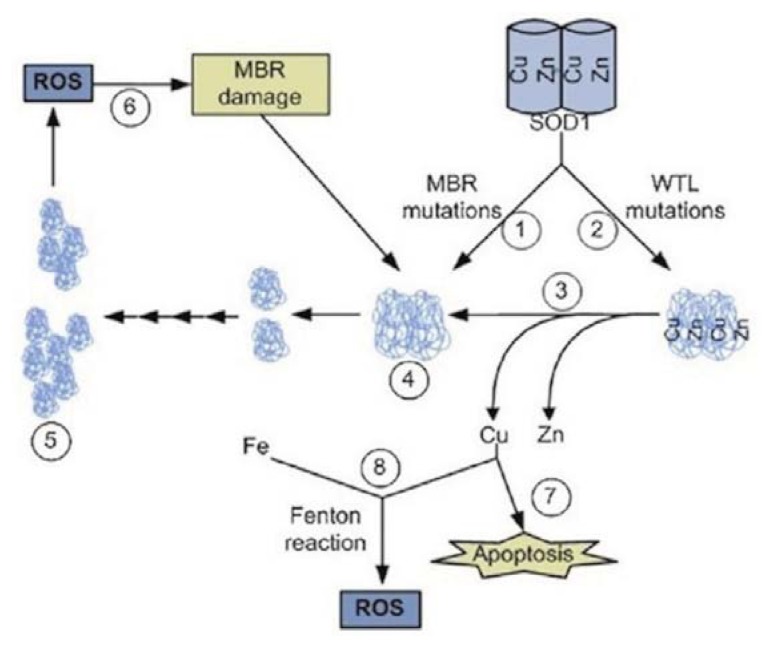
SOD1 structural changes and metal uncoupling in ALS: MBR and WTL-mutant SOD1 (1, 2) may promote metal uncoupling (3), protein aggregation (4, 5), oxidative damage (6) and apoptosis (7) (Source: Rivera-Mancía *et al.* [137]).

Interestingly, as SOD1 is Cu/Zn dependent, abnormal levels of both metals have been associated with ALS pathology in animal models [128–130]. Furthermore, structural changes of SOD1 may alter Zn and Cu levels. In fact, with regard to metal content [131] and position of the specific mutations, the fALS SOD1 proteins can be divided into two groups: (i) wild-type-like (WTL) mutant SOD1, in which metal content is similar to that found in the wild-type protein, and (ii) SOD1 with mutations at the metal-binding region (MBR) or at the electrostatic and Zn loop elements [132], associated with a deficiency in Zn and Cu content [131]. WTL-SOD1 mutants show high reactivity with hydrogen peroxide, producing site-specific oxidative damage which compromises metal binding, while MBR mutants may aggregate with no further modification [132,133]. Ultimately, the aggregation of both types of mutants may result in metal uncoupling and apoptosis (Figure 3).

Some hypotheses hold that SOD1 stability is dependent on its metal-binding state and the balance between normal and toxic SOD1 functioning depends on Zn binding at the active site of the enzyme [134]. In fact, in absence of Zn the catalytic reaction of SOD1 may run backwards, producing ROS [135]. SOD1 proteins, which have been oxidatively inactivated by reaction with hydrogen peroxide, lose their affinity for Cu and are more prone to aggregate than the wild-type protein [136]. Moreover, Cu released from Zn deficient SOD1 could potentially activate apoptosis [137] (Figure 3). As a consequence, Cu chelators, like antibiotics of the β-lactam type, penicillamine and *N*-acetylcysteine, have been proposed as novel therapeutic approaches in ALS, able to delay disease onset, improving motor performance and slowing disease progression in animal models [138–140], although evidences in humans are lacking.

### 3.5. Heavy Metals and other Chemical Compounds Implicated in Cigarette Smoking Neurotoxicity

Smoking has been associated with several neurodegenerative disorders. Prospective investigations found higher risk of dementia, also in association with other neurodegenerative diseases such as Parkinson’s disease, in smokers compared to non-smokers [141,142]. Recent epidemiological evidence has suggested a potential association between smoking and ALS, with some studies showing higher risk of ALS among smokers [14,15,143–145] and others not finding any clear association [146].

Several compounds have been identified in tobacco and cigarette smoking, including heavy metals, such as cadmium or lead, residues of pesticides used in tobacco cultivation [147] and formaldehyde [148]. The toxicity of cadmium as food and cigarette smoking contaminant or industrial pollutant has been well established [149]. With regard to cadmium involvement into neurodegenerative mechanisms, Huang *et al.* [150] have demonstrated that Cu/Zn SOD1 activity may be strongly inhibited by cadmium. In support of this, it has also been demonstrated that cadmium can replace Zn to reduce SOD activity [151,152]. Although heavy occupational exposure to cadmium has been associated to development of sALS [153], some epidemiological evidence found limited support to a possible involvement of cadmium or other trace heavy metals in the etiology of sALS [67–69].

Beyond the controversial role of lead exposure as risk factor for ALS, Weisskopf *et al.* [14] revealed for the first time in a large prospective study an increased risk for sALS with formaldehyde exposure, describing a strong trend with increasing years of exposure. Formaldehyde may have neurotoxic effects triggering oxidative stress, in part by reducing activity of SOD1 [154,155] and inducing mitochondrial membrane permeability [154,156], both mechanisms implicated in ALS [121].

Speculatively, it is possible that a gene-environment interplay able to hasten neurotoxic effects of cigarette smoking, potential susceptibility genes may be identified in paraoxonase genes cluster (PON) [157], encoding enzymes with antioxidative properties inhibited by cigarette smoking. More insight may also derive from exploring in ALS patients the relationship between mutations or polymorphisms of cytochrome P450 genes and cigarette smoking exposure, taking into account the recent findings about a possible role of this cluster of genes, encoding xenobiotic metabolizing enzymes whose mutations may modify the risk for cancer in smokers [158,159], as susceptibility gene for sALS [160].

### 3.6. Co-Exposure to Lead, Electrical Shocks and Electromagnetic Fields in “Electrical and Electronic” Workers

Several studies over the past two decades have shown that employees in electric industries, such as electricians, electric power installers and repairers, power plant operators, electrical and electronic equipment repairers, train drivers, telephone installers and repairers, and persons operating electrical equipment (*i.e.*, welders, carpenters, and machinists) may have an increased risk of developing ALS [7–10,13]. These categories of workers may be exposed to various potential neurotoxic agents, hypothesized to play a role in motoneurons degeneration, including heavy metals, especially lead [76], polychlorinated biphenyls [13], contained in insulating fluids, electrical shocks [7,8] and EMF [8–10,161]. However, with regard to electrical shocks, a systematic review of the literature of the last hundred years by Abhinav *et al.* [162] did not reveal a causal relationship between ALS and electrical injury, which may only trigger a non-progressive syndrome of spinal cord damage associated with the site of onset at the entry or exit point of the current. With regard to neurotoxicity of electromagnetic fields, a recent meta-analysis of seventeen epidemiological studies on the association between occupational exposure to extremely low-frequency (ELF)-EMF (*i.e.*, frequencies ranging from 3 to 3000 Hz) and the risk of ALS proved a slight but significant increase of the risk of developing ALS among ELF-EMF-related occupations [161]. However, it should be taken into account that public exposure to environmental ELF-EMF has rapidly increased in the last decades, and thus future research on the association between public ELF-EMF exposure level and ALS risk is needed to further clarify the potential role of this agent in ALS pathogenesis. Recent results derived from *in vitro* studies have revealed that prolonged exposure to ELF-EMF may induce cellular increase of reactive oxygen species with consequent impairment of antioxidant status, DNA damage and apoptosis [163,164]. Nevertheless, a potential link between ELF-EMF-related oxidative stress and ALS development has not been shown in SOD1 transgenic mice [165].

## 4. Pesticides

Pesticides have been largely examined as potential environmental risk factors for ALS because they are ubiquitous and involved in the pathogenesis of other neurodegenerative diseases, such as Parkinson’s and Alzheimer’s diseases [166,167].

The association between ALS and exposure to pesticides as a group has been explicitly evaluated in seven case-control studies [8,12,168–172] and one cohort study [14]. A recent meta-analysis of these studies revealed that ALS risk was associated with use of organochlorine insecticides (e.g., dichloro-diphenyl-trichloroethane), pyrethroids, herbicides, and fumigants but not with other pesticide classes [173]. Moreover, another recent meta-analysis of six peer-reviewed case-control and cohort studies [8,12,14,168–172,174,175] has supported the relationship of exposure to pesticides and development of ALS among male cases compared to controls [176].

Among herbicides, the organophosphate (OP) class, widely used in agriculture, accounts for one half of the total pesticide consumption yearly in the USA [177]. Chronic exposure to OPs has been found to induce progressive brain damage by irreversibly inhibiting acetylcholinesterase, resulting in excessive stimulation of cholinergic receptors and excitotoxicity [178]. Several studies, investigating the potential association between exposure to OPs and risk of developing ALS, have emphasized the role of some predisposing genes in this pathogenic pathway. Specifically, Morahan *et al.* [179] showed that impaired ability of sALS patients to detoxify pesticides, heavy metals and chemicals could be related to polymorphisms in metallothionein family of genes, encoding metal transcription factor-1 and glutathione synthetase.

In this regard, there is growing interest about the pathogenetic role of paraoxonase genes cluster [157,180], of which PON1 is most intensively studied, codifying for the A-esterase paraoxonase-1 able to detoxify the prototypical OP, paraoxon. Given that PON1 hydrolyses OPs and ability to detoxify them is largely determined by its different variants [181], it is assumed that the individual risk of developing ALS after exposure to OPs could grow in case of genetic mutations and suboptimal body concentration of PON1 [182,183]. In particular, mutations that impair the ability of PON1 to detoxify OPs could increase the sensitivity of patients to OPs and potentially lead to development of ALS [184–187]. However, with regard to the investigations on a causal link between exposure to OPs and mutations in the PON1 gene, epidemiological and animal studies provided conflicting results [157,180,188,189]. In particular, Ticozzi *et al.* [157] sequencing PON1 gene in 260 fALS, 188 sALS and 188 control subjects and PON2 and 3 genes in 166 fALS patients identified eight coding sequence mutations in the PON genes of nine fALS and three sALS cases but not in controls, suggesting that mutations in the PON genes may be involved more significantly in fALS than in sALS. Additionally, a recent meta-analysis of all published data showed that actual results failed to detect an association between polymorphisms in the PON locus and susceptibility to sALS [180]. Furthermore, van Blitterswijk *et al.* [189], assessing the frequency of PON variants in 1118 sALS patients, 93 fALS patients, and 1240 control subjects of Dutch ancestry, detected no significant differences in mutational burden for rare variants or in allele frequencies of PON polymorphisms between patients and control subjects, concluding that mutations or polymorphisms in PON do not contribute to ALS susceptibility. Nonetheless, there is always a strong support in favor of a causal link between OPs exposure, some paraoxonase variants (Q and R allozymes) and Gulf War Syndrome [29,30].

## 5. Concluding Remarks and Future Perspectives

The mechanisms of motor neuron degeneration in ALS are steadily being discovered. The heterogeneity of several possible etiologic factors able to induce neurodegeneration in ALS, from mutations of genes encoding ubiquitous proteins with role of scavengers or modulators of gene transcription to yet uncertain environmental factors, underlines the multifactorial nature of ALS. Therefore, given that gene-environment interplays have been proved relevant for all environmental causes of ALS, the effective role of environmental risks factors for ALS pathogenesis should be essentially explored by further investigations at both epidemiological and genetical levels, whilst cellular and animal models may provide useful tools to explore the underlying biochemistry of motor neuron degeneration. Lack of a consistent literature which considers the contribution of multiple susceptibility factors acting together in ALS pathogenesis might explain the detection of conflicting results about the potential role of environmental toxins. Moreover, additional prospective studies should focus on specific target populations and assessment of possible pollutant sources, using environmental modeling techniques and geographical information systems. In fact, the recent availability of powerful tools such as population-based ALS registries for case ascertainment and clustering detection might contribute to provide useful information and interesting clues for more insights into the etiopathology of the disease [190].

On the basis of the reviewed epidemiological and experimental evidence, cyanotoxin BMAA, Se and pesticides exhibit some biological plausibilities, such as triggering factors or causal agents for ALS, and only PON genes have been found to play a possible role in mediating the effects of environmental toxins in inducing ALS onset. The few or conflicting results about involvement of Pb, Hg, electrical shocks and EMF into ALS pathogenesis may be probably due to methodological limitations of the studies on these topics, such as low statistical power, exposure misclassification, and still inadequate control of confounders.

**Figure 4:**
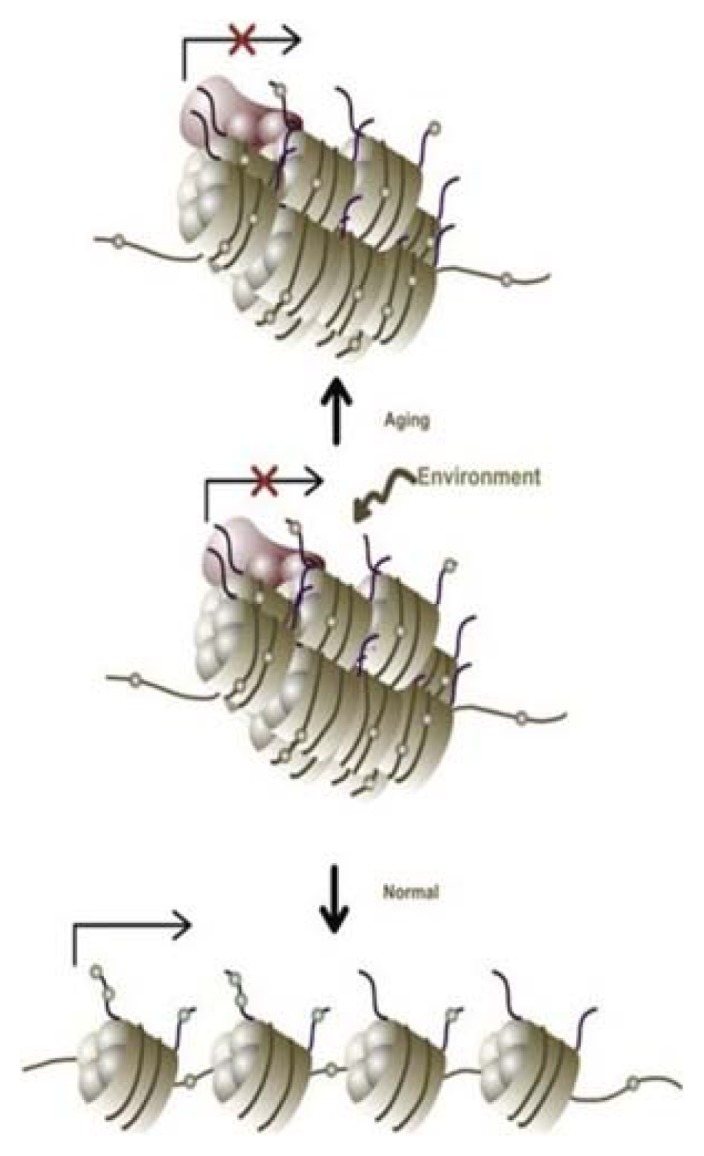
Loss of the normal genetic plasticity with aging: if a gene undergoes environmental-dependent epigenetic change, the ability to respond and revert this modification could be reduced because of aging, and the gene may be repressed (Source: Marques *et al.* [192]).

Remarkably, in the last decade, the field of epigenomics has emerged, revealing that DNA modifications, including DNA-bound histones, DNA methylation, and chromatin remodeling, which may depend from environmental clues, such as lifestyle, diet and toxin exposure, also provide levels of gene regulation and alter gene expression. Epigenetic factors are probably much more suited than genetic factors to explain disease onset and progression in ALS, since aberrant epigenetic patterns may be acquired throughout life [18,191]. One hypothesis is that environmental life exposures result in a failure to maintain epigenetic homeostasis in the CNS, which in turn leads to aberrant gene expression, contributing to CNS dysfunction and in some cases the development of ALS. This epigenetic modulation might be quite similar to that described during aging and associated with the loss of the normal phenotypic plasticity to external environmental signals [192] (Figure 4).

Future studies are needed to investigate this intriguing pathogenetic mechanism, and eventually to identify new therapeutic targets.
